# An exploration of young people’s, parent/carers’, and professionals’ experiences of a voluntary sector organisation operating a Youth Information, Advice, and Counselling (YIAC) model in a disadvantaged area

**DOI:** 10.1186/s12913-022-07800-1

**Published:** 2022-03-23

**Authors:** Shaima M. Hassan, Joanne Worsley, Lisa Nolan, Nicky Fearon, Adele Ring, Jane Shelton, David McEgan, Farheen Yameen, Esmaeil Morasae Khedmati, Cecil Kullu

**Affiliations:** 1 Department of Primary Care and Mental Health, Institute of Population Health Sciences, University of Liverpool, Waterhouse Building, Brownlow Street, L69 3GL Liverpool, UK; 2 NIHR Applied Research Collaboration North West Coast ARC NWC, Liverpool, UK; 3 Liverpool Clinical Commissioning Group, Liverpool, UK; 4 Mersey Care NHS Foundation Trust, Liverpool, UK; 5 Business School, University of Exeter, Exeter, UK

**Keywords:** Young people, YIAC model, Access, Engagement, Mental health, Wellbeing

## Abstract

**Background:**

The present evaluation explored young people’s, parents/carers, and healthcare professionals’ perceptions of the Youth Information, Advice and Counselling (YIAC) model operated by a voluntary sector organisation in North West England. With an aim to understand the key components that contribute to enhancing the success of the YIAC model.

**Method:**

Semi-structured interviews and focus groups with young people, parents/carers, and healthcare professionals were conducted. Data were analysed using thematic analysis.

**Results:**

Five main themes were identified from the data: 1) Accessibility and flexibility; 2) Non-clinical model and environment; 3) Staff; 4) Partnership working; and 5) Promotion of positive mental health and wellbeing.

**Conclusion:**

Findings highlight the importance of non-clinical, community-based, ‘one-stop-shop’ hubs for young people in disadvantaged areas. The key components highlighted as facilitating access and engagement include: opportunity to self-refer, choice of location, timely provision of support, non-clinical environment, age appropriate services, a non-hierarchical workforce, inclusive support for family and carers, a focus on wider, often social, issues, and collaboration with partner organisations. These findings suggest that early support hubs for young people’s mental health should have consistent, long-term funding and should exist in every local area.

**Supplementary Information:**

The online version contains supplementary material available at 10.1186/s12913-022-07800-1.

## Background

One in 10 young people in the United Kingdom (UK) suffer from a diagnosable mental health problem [1]. Most mental health problems emerge between the ages of 12–24 years [2, 3], with the majority of adults living with mental health problems having first experienced mental health difficulties before the age of 18 years [4]. Young people from disadvantaged backgrounds are three times more likely to develop mental health problems than those from more advantaged backgrounds [3], and childhood disadvantage that compromises health in adult life (and contributes to mental health problems) often remains undetected until later in life [2, 5]. Young people can face at least a decade between first becoming unwell and seeking or receiving support [6]. Thus, despite the prevalence of mental health problems, young people in this age group are still most likely to experience multiple gaps, a dramatic culture shift, and lack of continuity of care between youth and adult services [7].

There are many barriers to seeking support for young people with mental health problems, such as lack of awareness of services and stigma surrounding mental health, which can all influence the extent of help seeking and consequently the timeframe to receipt of support by young people [8]. More common mental health problems, such as depression and anxiety, experienced by young people tend to increase social withdrawal, making it even more likely that they will not seek support [9]. Other barriers to access can include family members; often parents or carers are the “gatekeepers” for young people’s access to mental health services. Parents and carers also experience a range of barriers when trying to access services for their children, which include issues such as referral criteria, but also include lack of knowledge and understanding of mental health services and how to access them [10].

Specialist Child and Adolescent Mental Health Services (CAMHS) are NHS-funded statutory services in the UK which assess and support young people with mental health difficulties [11]. Many services that provide specialist mental health care have long waiting lists [12, 13]. Young people have expressed major concerns about waiting times to access mental health support, which for some was found to contribute to a deterioration in their mental health and/or to them reaching crisis point [14]. In addition to long waiting lists, reports have also consistently noted additional difficulties that young people face in accessing CAMHS, such as an increase in the number of referrals and thresholds for entry [15].

Nevertheless, as such provision continues to adopt a clinical approach, these services are not designed to support the wider needs of young people or encourage engagement [16]. According to Lerner (2005, p.5) [17], rather than seeing young people as ‘broken’ and ‘in need of repair’, youth should be seen as ‘resources to be developed’. This perspective emerges from the individual context model of relationship development theory [18]. In line with this perspective, service providers are encouraged to draw on young people’s strengths and coping capacities, as well as the physical and relational resources available to them [19, 20]. Such approaches require the development of trusting relationships, enabling practitioners to understand and meaningfully respond to young people’s contexts, risks, and resources. In fact, the quality of relationships that professionals form with young people may be as important in determining outcomes as are intervention modalities or programme elements [21].

In 2015, the ‘Future in Mind’ report [4] provoked a national inquiry into CAMHS, and highlighted the importance of promoting, protecting and improving young people’s mental health and wellbeing. This report recognised the benefits of addressing the unmet need for services in this age group through the provision of integrated Youth Information Advice and Counselling Services (YIACS), led by Youth Access nationally. This approach emphasises the importance of whole systems change, integrated support, and services that provide support to young people up to the age of 25 years [4]. The main aim of YIACS is to offer an integrated approach to addressing young people’s health and wellbeing concerns, through a unique combination of non-clinical community-based prevention, early intervention and crisis work. This approach reflects the NHS Five year forward view on prevention through community engagement and integration of services, and the NHS Long Term Plan’s commitment to universal personalised care [12, 13, 22]. Of importance, Youth Access reports indicate that voluntary sector services are often more acceptable to young people and are user focused carrying less stigma than statutory services [23]. There are currently a number of YIACS across England, which share common features including young person-centred care and support addressing multiple and complex needs, eligible to a wide age range of young people, flexible free access to service, and multidisciplinary teams providing wrap around support.

The present evaluation explored young people’s, parents/carers, and healthcare professionals’ perceptions of the Youth Information, Advice and Counselling (YIAC) model operated by a voluntary sector organisation in North West England. In particular, young people’s experiences of accessing and engagement with the service, and the processes and mechanisms that facilitated or hindered access and engagement, were explored.

## Method

### The Youth Information, Advice, and Counselling (YIAC) model

The voluntary sector organisation operating a YIAC model, commissioned by a Clinical Commissioning Group (CCG) in the North West of England, provides information, advice and counselling for young people aged 14–25 years. This model is led through Youth Access which is a national organisation that advocates for services that support young people’s needs and focuses on incorporating therapy-based services with those that support the wider issues affecting young people’s mental health. This includes easy access to services, appropriate support available in one place, and a young person-centred approach to care. The YIAC model facilitates access for young people via professionals, family referrals and self-referral across three community hubs in a disadvantaged city, which has a registered practice population of 177,316 that are aged between the ages of 0 to 25 years. During 2017–2018, 1520 children and young people accessed the community hubs [24].

The current YIAC model delivered by the voluntary sector organisation offers a wide range of services that deliver social, psychological and health support for young people and their families. The service takes a collaborative, whole family, needs led approach, in working with partner agencies across children and adult services. Support/services provided include: health advice and guidance, drop-in facility, monthly GP drop-in, substance misuse support, LGBTQ + service, neurodevelopmental support, parenting/family-based support, multi-modal counselling, psychological support and psychosocial education. A young person’s first point of contact with the service is an Information Advice and Guidance (IAG) worker who undertakes an initial assessment of their needs and offers them a choice of interventions to support their identified needs. This may involve young people accessing one or multiple interventions in voluntary sector organisation or other partner organisations.

### Ethical consideration

The project was deemed a service evaluation according to the local NHS Research & Development department. Approval for the project was obtained from the Liverpool Clinical Commissioning Group. This study protocol, participant information sheets, topic guide and consent forms were reviewed by an NHS Clinical Commissioning Group (CCG) Research & Development department as a service evaluation. This evaluation complied with the CCG Health Research Authority (HRA) guidelines, which promotes the interests of patients and the public in health and social care research.

### Recruitment and participants

Posters and leaflets (containing brief study details and research team contact details for expression of interest) were distributed across child and adult mental health providers, primary care services, youth and community providers, and third sector organisations. Potential participants who were interested in taking part in the evaluation contacted the project intern (LN) to express their interest. Potential participants were provided with the participant information sheet and consent form to read through before taking part in the study. The intern discussed with participants any questions they had about the evaluation to ensure they fully understood what their participation would involve. Parents and professionals provided informed consent before attending a scheduled interview or a focus group. Although young people involved in the study were above the age of 16 years, and were able to provide informed consent, those who expressed an interest in taking part were asked to share the details with their parent or carer who were asked to make contact with the project intern (LN) to confirm they were happy for their child to participate. Practitioners from the voluntary sector organisation were available, if required, to support any young person or parent who became distressed during the interview process.

In total, 36 participants took part in the evaluation: 24 young people aged between 16 and 25 years who were accessing the voluntary sector organisation, five parents/carers who had children who were accessing support at the voluntary sector organisation, and seven healthcare professionals who had either referred a young person to the voluntary sector organisation or worked alongside the organisation participated in the evaluation. A £10 gift voucher was offered to young people and parents/carers in recognition of their time in participating in the study. A total of 11 in-depth one-to-one interviews (four young people, three parents and four healthcare professionals) and four focus groups were conducted: two focus groups with young people, one focus group with parents, and one focus group with healthcare professionals.

### Topic guides

Semi-structured one-to-one interviews and focus groups with young people, parents/carers, and healthcare professionals captured their experiences of accessing and engaging with the voluntary sector organisation including how they got involved and how they perceived it to have helped them. Topic guides were co-designed by young people, public advisers (JS, DM & FY), academics, and a CAMHS participation worker. The CAMHS participation worker reviewed the topic guide for children and young people to ensure that all questions were age appropriate. Two of the public advisors were parents of children and young people who had accessed CAMHS and one was from the ethnic minority community. Public advisors ensured that questions were relevant and meaningful for parents and carers. Interview and focus group topic guides included questions such as ‘Can you tell me about the services you have accessed in voluntary sector organisation and what you hoped to gain from accessing this service?’, ‘What did you like about the service?’ and ‘What did you not like about the service?’ (see additional file 1).

### Data collection

Interviews and focus groups were conducted by the project intern (LN) and one of the public advisers (JS, DM & FY) who had no prior relationships with the participants. All interviews and focus groups were conducted during June – July 2018 in community hubs, except for one professional focus group and one professional interview, which were conducted within the professionals’ place of work. Focus groups lasted approximately 60 min. Interviews lasted approximately 30 min. Interview data were collected until no new, or repetitive, information emerged from the interviews. Data collection concluded once the saturation of themes occurred [25]. No new information emerged after ten interviews. All interviews and focus groups were audio recorded and transcribed verbatim by a university professional transcription service. Transcripts were reviewed and checked for accuracy by the project intern (LN).

### Data analysis

Data were analysed using the thematic analysis procedure outlined by Braun and Clarke [26]. Initially, the project intern, alongside one of the public advisers, went through a process of reading the transcripts, highlighting and discussing meaningful narratives. The project intern engaged with researchers throughout the data collection and analysis process to reduce research bias. All transcripts were also coded independently by two researchers (JW and SH) who had no prior relationships with the participants. The research team met frequently to discuss and refine new codes. As the research team had different backgrounds, this enabled a thorough discussion of different perspectives, thereby reducing individual biases. As the initial themes captured by coding were refined during discussions with the wider team, this ensured that the final themes were not just the personal interpretation on one team member.

### Public involvement

Three public members were involved in the study as public advisers. Public advisers were all carers for young people who had experience of mental health care services but not specifically the voluntary sector organisation [YIAC]. Public advisers were involved at all stages of the study, ensuring that a public perspective informed the research process throughout. For example, advisers had instrumental roles in data collection and interpretation of research findings. Public advisers received training in qualitative research methods to support their role in co-conducting focus groups and interviews.

## Results

The evaluation drew on thematic analysis of semi-structured interviews and focus groups with young people (*n* = 24), parents/carers (*n* = 5), and healthcare professionals (*n* = 7). Five overarching themes were identified from the data: 1) Accessibility and flexibility; 2) Non-clinical model and environment; 3) Staff; 4) Partnership working; and 5) Promotion of positive mental health and wellbeing.

### Accessibility and flexibility

Accessibility is a key feature of the YIAC model and information from participants supports this proposition. Key to participants’ perceptions of accessibility were options to ‘self-refer’, the location of the three hubs, and the flexible appointment times. The voluntary sector organisation as a service was described as “*incredibly easy to access*” (Professional 3, NHS worker) as “*people can just walk in off the street*” (Professional 1, Third sector organisation). Although young people can be referred by their GP, the process of referral at the organisation is unique as young people are also able to self-refer. Self-referral is important and often encouraged by professionals from other services in order empower young people: “*I try and encourage self-referral to empower people… The belief is if we do that for them and it’s not owned by the young person or by the parent then because there’s no ownership they might just let it fall”* (Professional 1, Third sector organisation).

Location was also an important factor in participant’s appraisal of service accessibility, with some preferring the central hub over the North and South hubs: “*It’s really accessible with it being in the centre near enough the centre of town*” (Young person 3) and “*I just prefer to come to the central one because the other 2 aren’t really accessible to where I live*” (Young person 1).

Flexibility of appointment times was also viewed as an important factor in accessing the service as this enabled young people and parents with school, college, or work commitments to select appointments that suited their day-to-day lives: “*they’ll let you choose your appointments. They work around you in case you’re in college or work*” (Young person 2). Many young people in this study highlighted that group sessions were held at reasonable times: “*the timings of the groups really suited me because they were at the time I was still in school so they were straight after school*” (Young person 4).

Waiting times varied with some young people accessing support relatively quickly whilst others encountered long waiting lists. Longer waiting times were reported for counselling and psychological services, whilst waiting times were shorter for LGBTQ + provision and Information, Advice and Guidance: “*I asked to see a counsellor…My waiting time was originally going to be about 3 months but I think it was cut down to about 1 month*” (Young person 1) and “*It [referring to waiting list for counselling] was about 6 months*” (Young person 2).

### A non-clinical model and environment

Healthcare professionals acknowledged the importance of a social model: ‘*it does take much more of a social model around mental health because I always think you need to [explore] what are the conditions in society which are making the person experience mental distress*’ (Professional 1, Third sector organisation) and ‘*I think there’s something about the model that happens in YPAS in terms of it helps people. You’ve got… information, advice and guidance that maybe helps people with some of the practical issues which brings down some of the stresses… Then the therapeutic work seems to be pretty helpful in terms of I suppose taking that more normalising approach to being able to manage their mental health. It’s not diagnostic and here’s lots of medicine. I think for a lot of people that’s exactly what it’s about. It’s about learning a bit more about yourself, learning a bit more how to tolerate it or maybe move on from some difficult or traumatic experiences that you’ve had*’ (Professional 3, NHS worker).

Young people and parents valued the non-clinical design of the community hubs: “*it’s really welcoming it’s not clinical of any sort. I’ve accessed other places be it in hospital or GP surgeries or whatever and it’s a lot more with all the white around the room, it can feel quite daunting a bit robotic. But now in [organisation] you got a real cosy feel, family and generally just welcoming*” (Young person 1). Participants described this environment as important for engaging with YIAC services and enhancing the therapeutic relationship – comparing this approach to their experience of other services they had previously accessed prior to their involvement with the voluntary sector organisation: “[referring to other services] *the buildings dreary, it’s dark, it’s not a particularly nice place I don’t think to go and then my experience with seeing a psychiatrist in there was being led through little corridors into this room that was just a mess and for a kid with sensory issues who struggles to have a rapport with anyone that put him on I think on a back foot before anything would of even started*” (Parent 1) and “*it [the voluntary sector organisation] was a lot more comfortable. I’m quite nervous around hospitals*” (Young person 4). In contrast, the welcoming environment of the community hubs may have helped to allay young people’s anxieties about accessing support from the voluntary sector organisation, as one young person described when reflecting on visiting the central hub for the first time: “*I’m comfortable here and I feel welcomed, makes me feel warm on the inside when you first come in not shaking like a leaf*” (Young person 1). One professional also commented on the way in which the environment influenced the dynamics of their sessions: “*I was doing a session with someone and I was in a yellow room with decorated from in-service chairs and it was a completely different session then it probably would have been if they’d been in here, which is all quite grey and sterile and clinical*” (Professional 3, NHS worker).

Professionals in this study also valued the informal, non-clinical environment at the community hubs, emphasising the sense of positivity that contributed to the welcoming atmosphere of the hub: “*it’s one of the things that always strikes me when I go in how there’s lots of positivity and positive messages and it’s also busy but very welcoming and it just feels like a co-created safe young people’s space*” (Professional 3, NHS worker). Another important feature of the environment noted by professionals was its age-appropriateness: “*you can go out and do your assessments for people that are under 25 in the hubs so you’re seeing them in maybe more age appropriate environments*” (Professional 2, GP).

### Staff

Young people valued the non-hierarchical structure of the service and emphasised the importance of this for equality: “*you don’t get any sense there’s a hierarchical structure here at all, everyone is treated equally. Young people are at the forefront of the services that [voluntary sector organisation] administer if that right word, provide and their views are always taken into account at every level*” (Young person 1). This approach was also important for brokering early conversations, with young people describing how they felt comfortable opening up to the staff at the organisation about their difficulties: “*even the staff you don’t know too well they all seem to be like very well trained or just genuinely nice people to where you kind of feel like you can talk to them like straight away even if you’ve only met them for like 10 min*” (Young person 3). Staff were often described by participants as friendly, supportive, and understanding, which enabled young people to feel comfortable: “[the staff are] *the most understanding and friendly and they actually knew how to like talk to me and didn’t talk down to me about stuff*” (Young person 3).

For many young people, the opportunity to talk to others who share similar experiences was particularly important and was a noted benefit of attending the service: “*the people that they have here are mostly LGBT themselves, so they can give an older perspective on things to tell the younger people that its actually going to get better*… *it was just like meeting someone who’s actually trans was really helpful*” (Young person 3) and “*He was the first trans person I’d ever met… he said we’re going to do six week of informal counselling sessions where we just discuss your gender, how to express yourself, and questions you feel comfortable with. 18 weeks later I was actually at the gender clinic for my first appointment so they really helped*” (Young person 4). This opportunity to interact with similar others, either professionals or other young people, was noted by participants to be an important aspect of care often missing in other services: “*I attend [name of voluntary sector organisation] because I’m transgender and the service I was attending wasn’t very helpful, there was just definitely a need for people who are going through the same things as me and needed some friends who were like that*” (Young person 3).

In line with this, professional participants also praised the staff, recognising that when a young person is in distress, often all they need “*is an independent person to speak to who is comfortable and skilled at talking to young people who can respond to the cues who’s aware of the whole psychosocial nature of wellness and wellbeing*” (Professional 2, GP). This was described as one of the key elements of the organisation: “*the selling point is you will talk to someone who works with young people day in day out*” (Professional 2, GP). Parents also found the staff at voluntary sector organisation to be empathic and understanding: “*so understanding and it’s like she really gets you*” (Parent 2) and “*the first time of meeting her I was dead comfortable and she really made me feel like better*” (Parent 1). The importance of this approach for parents became apparent as they described feeling as though they were finally being ‘*listened to*’, ‘*not being pushed from pillar to post’* and ‘*supported as a whole family’* at the voluntary sector organisation. For example, “*I felt like I was getting passed from pillar to post and to be really honest with you the only progress I made was through [name] the lady from [organisation], and I just felt like I was getting listened to as a parent*” (Parent 3).

### Partnership working

The YIAC model facilitates access to wider support provided by partner organisations. These included services in a city in the North West of England that run specific courses supporting young people around employment: “*I’m on a course with [name of organisation] in a course that involved the NHS and going round all the different Trusts experiencing what it’s like to work in the hospital.*” (Young person 1) and “*they’ve also put me in touch with [name of organisation] that help me to potentially get a job in the future*” (Young person 1). Parents are also signposted to organisations that offer practical support including information about available financial resources: “*she put us in touch with I know it’s not really a service but the [name] were brilliant, because I didn’t know about the DLA [Disability Living Allowance] and things like that having worked all my life I wouldn’t of even considered claiming, I didn’t even know I would be able to claim for anything, I was like why would I I’ve always worked*” (Parent 1). Also, the voluntary sector organisation was able to signpost parents to specialist support for particular difficulties such as autism or attention-deficit hyperactivity disorder.

The professionals in this study working at other organisations in the North West of England viewed the voluntary sector organisation delivering YIAC as a strong partner organisation with connections across the city to refer children and young people to for support: “*as a strong partner organisation to [name], a really good place to be able to refer children and young people and their families to assess mental health support, information, advice, and guidance*” (Professional 1, Third sector organisation).

### Promotion of positive mental health and wellbeing

Young people in this study spoke about how the organisation had helped to improve their mental health and wellbeing including: (i) gaining a sense of purpose; (ii) feeling optimistic about the future, (iii) developing positive attributes such as building confidence; (iv) reduced symptoms of anxiety; and (v) expanding their social circle by cultivating friendships. For example: “*[The] organisation has certainly helped me come to terms with myself in as such it’s given me a sense of purpose coming here and doing all the different events and activities I do with them and also the work I do for young ambassadors*” (Young person 1) and “*they’ve helped me socially to come out to be confident as a young person in myself and to trust others. Helped me to be less anxious through a little bit of counselling and the groups. I wouldn’t be able to recognise myself now from then*” (Young person 4). Young people in this study also felt that the organisation enabled them to feel more optimistic about their future: “*I just became a lot more happy in myself and a lot better about the future and like before I’d be like ‘oh I’m not even going to make it college’ and wow going to college this year wow wow wow things like that they’ve just helped me out*” (Young person 3). Young people often spoke candidly about developing as a person and acquiring new positive attributes: “*it’s certainly helped me in life and it’s got a special place in my heart because it’s helped me it’s helped develop me into the person I am today, a lot more confident*” (Young person 1), and “*they kept me on my feet. I wouldn’t be who I am or where I am today without [name of organisation]*” (Young person 2).

Before attending the voluntary sector organisation, many young people in this study lacked confidence and found it difficult to socialise with others their age: “*I used to just get very nervous around other teenagers like I couldn’t hang out around people my age at all and then I start going to the group and I was like ‘oh teens aren’t that scary’ and it just helped my confidence a lot and I think I came here wanting to feel more confident about some things and definitely have*” (Young person 3); “*it’s also helped to develop my social skills as well given that I’m on the autistic spectrum so previously I was a bit of a closed book when I first attended*” (Young person 1) and “*I’ve met new friends, gained more confidence*” (Young person 2). Attending the voluntary sector organisation enabled young people to meet and develop friendships with similar others: “*I was desperate to meet people who were like me because in my school I was the only one that was transgender*” (Young person 4). Opportunities to interact with similar others was described as important for young people to develop a shared understanding and mix with people that had shared common interests: “*I just came here and met more teenagers that are like me and have common interests and they’ve motivated me to pursue hobbies that I actually like just nerdy stuff and they’ve just helped me become me*” (Young person 3). Healthcare professionals also shared similar views. For example, ‘*I’ve seen them [referring to YPAS] be really really helpful for a lot of young people. I think particularly when people are struggling to make sense of their current mental health and their current situation, their supportive relationships do that either therapeutically or I think the other thing that I’ve seen young people getting a lot from is actually the service itself in terms of its drop in and its groups… I think what you need is to have somebody to talk to about where you’re at and actually be able to form some social relationships and move forward…I’ve seen people that have come and been in quite a distressed state actually sat with other young people having an alright time’* (Professional 3, NHS worker).

In a similar way, parents valued the opportunity to attend groups at the voluntary sector organisation where they could meet and interact with similar others who had a shared understanding of the difficulties they faced and formed part of their support network: “*support in the sense of like now coming to that wellbeing group you’re not alone, there are other parents going through exactly the same thing and it’s heart breaking when you hear the stories and it’s nice that you’re not travelling that journey alone”* (Parent 2). Participants highlighted that attending these groups also provided an opportunity for them to have a small break from their daily routine and usual commitments: “*I look forward to Friday because Friday is my day when I can just escape and I come here and there’s other people that get what you’re going on about*” (Parent 1) and “*I think this wellness group that they’ve put on has been great this relaxation things we do all different things and that it’s been really has helped me and it means me getting out. Because and it sounds horrible I’m stuck with him because I can’t leave him on his own*” (Parent 1). In addition, the groups at the organisation also equipped parents with coping strategies and new skills: “*I’m doing this well-being course. So like it’s good because it’s giving us strategies because I don’t sleep very well so it has given us strategies of like how to a guy called [name] is teaching us how to like just relax and about breathing techniques and mindfulness*” (Parent 3).

## Discussion

The present evaluation explored young people’s, parents/carers, and healthcare professionals’ perceptions of the Youth Information, Advice and Counselling (YIAC) model operated by a voluntary sector organisation in North West England. This included exploring young people’s perceptions of the YIAC model, their experiences of accessing and engagement with the service, and the processes and mechanisms that facilitated or hindered access and engagement.

The voluntary sector organisation operating the YIAC model, from participants’ perspectives, offers a non-clinical and age appropriate environment providing easily accessible information, advice and support, and is unique due to the inclusive nature of their offer. These perspectives reflect the ethos of youth-based approaches to healthcare provision for young people experiencing mental health difficulties, and the NHS Long Term Plan commitment to move towards person-centred, age appropriate services for young people [6, 17, 18, 27]. Our findings highlight the key components that facilitated effective engagement of young people and their families and enhanced their access to appropriate information, advice, and support. These components include: opportunity to self-refer, timely access, choice in location, non-hierarchical service structure, non-clinical environment, a focus on wider social issues, a non-stigmatising setting, age appropriate, support that was inclusive of the wider family, and partnership working. By facilitating better access and engagement, the voluntary sector organisation reflects the Future in Mind recommendation to establishing plans promoting, protecting and improving young people’s mental health and wellbeing [4].

The YIAC model promoted and eased the process of advice and support seeking for young people and their families. For example, the importance of having the option to self-refer as a key mechanism for facilitating participants’ engagement with the YIAC service was highlighted. This aspect of the service is important in terms of agency and empowerment. Open access to community-based hubs enhanced choice and facilitated access to services at the point of need rather than having to wait for referral. It should be noted here, however, that the voluntary sector organisation delivering the YIAC model encountered significant funding cuts during 2017. One impact of funding cuts was longer waiting times for some elements of the service – with many participants referring to long waiting lists and the strain the service was under due to funding cuts. Although this evaluation took place when the service had experienced a significant funding reduction, participants still found the service to be accessible.

There is a long-standing concern that current service structures do not effectively meet the needs of young people with mental health problems [28–30]. The voluntary sector organisation aims to address this by moving away from the conventional model of care delivered within clinical settings that often perpetuate traditional hierarchical structures that impact interactions between provider and service users. This is particularly important when providing care for children and young people who experienced social challenges, which may lead to them struggling to interact with others [31]. One study highlights how young people with mental health difficulties reported that their reason for delaying or not seeking professional help was because they did not feel confident to express their feelings, emotions and thoughts [8]. Participants in the current study reported that providing a community mental health service outside of traditional clinical settings and with access to a multidisciplinary team (therapists, youth workers, social workers, parenting practitioners, sessional workers, wellbeing workers, IAG workers, volunteers and students) contributed to their engagement with the service.

Clinical provision is often set up around a medicalised model of care that encourages clinicians to operate within their specialism rather than adopting a more holistic approach to care provision [32]. Consequently, it is of note that professionals in the current study reported noticeable benefits of providing care within a community, rather than clinical, setting. This included aspects of the physical environment, which offered a more relaxed and comforting atmosphere that was thought to facilitate the expression of difficult emotions and current needs by young people and understanding of those needs by experts. The provision of services within environments experienced as more engaging by both young people and health professionals is an important step in responding to the Department of Health pledge to improve health outcomes of young people [33]. Indeed, previous research [34] demonstrates that youth counselling provided through YIAC services shows similar clinical outcomes to those reported in CAMHS or school-based support. In line with this, young people who took part in this evaluation reported general improvements in their mental health and wellbeing. High levels of service satisfaction were also expressed by young people. What was unique about the voluntary sector organisation was the focus on the wider issues young people were experiencing such as issues surrounding sexual identity. Young people reported that having the voluntary sector organisation workers who had experienced similar issues helped to build their confidence, enabling them to feel understood when expressing their needs. The non-hierarchical structure was important in helping to broker conversations between service staff and young people in a non-stigmatising setting. Positive relationships with staff provided a safe context for young people, enabling them to express themselves and feel heard. To build these trusting relationships, staff respected young people and their beliefs, including respect for gender identity and cultural or religious beliefs. In turn, these relationships created a space for engagement and empowerment.

Young people and parents in this evaluation reported that the voluntary sector organisation gave them the time and space to engage with a service that strongly incorporates a whole family concept in its approach. Parents reported initially feeling alone and experiencing stress (which included social issues) when dealing with their child’s mental health issues. However, accessing the voluntary sector organisation was considered to have provided support not only for the needs of their child but also for their needs as parents. This is an important element in providing care for young people, as parents are not only instrumental in recognizing a child's mental health issues but also in building their confidence in interacting with health care services including making decisions about when to seek support and how to obtain treatment [10].

Awareness of locally available resources help in addressing the social factors (such as poor living conditions, low family income and poor family support), which contribute to mental health issues faced by young people and their parents [35]. The strong partner ethos, were collaboration with other services enhanced the arrangement and delivery of appropriate care for young people, was a component of the service that was valued by participants. This is an important element of the model given that lack of collaboration between organisations can have an adverse effect on young people and their family’s experience of care, often leaving young people without timely appropriate support [36, 37].

### Strength and limitations

This study explored the approach of YIAC in addressing the mental health needs of young people in a disadvantaged area. The qualitative approach to enquiry in this study was important enabling us to attune to the unique experiences of young people, parents and health professionals, providing key insights about what they considered to be the successful components of this model. These findings suggest that early support hubs for young people’s mental health should have consistent, long-term funding and should exist in every local area.

However, we acknowledge that our data only provides a snapshot of people’s experience of the service. A more longitudinal approach following young people from their point of entry into the service and their subsequent journey within the service, may have helped us to gain a greater understanding of the wider and more long-term impact of the YIAC model. This is to also include a quantitative approach to include different demographics that can impact young people’s engagement and access to mental health services. Last, it is important to acknowledge biases in sampling. As the sampling was not purposive, future research in this area should be conducted on purposive samples.

## Conclusion

Key components of service quality include positive relationships with caring adults, the development of life skills, and opportunities for youth engagement and empowerment [38]. Processes that support engagement helped to improve outcomes for young people and their families. Particular aspects that facilitated engagement were the opportunity to self-refer, choice in location of service delivery, timely provision of support, age appropriate services, a non-hierarchical workforce, inclusive support for family and carers, a focus on current mental health issues and collaboration with partner organisations. It will be important in the future to undertake a longitudinal evaluation to understand the extent to which health benefits are sustained over time.

## Supplementary Information

**Additional file 1.**

## Data Availability

Qualitative data extracts are presented in the article to support the findings. The original transcripts are not available to the public as they may contain information that could compromise the confidentiality and anonymity of the participants, but are available (limited) from the corresponding author on reasonable request.

## Abbreviations

YIACS: Youth Information Access and Counselling Service
CCG: Clinical Commissioning Group
PPP: Partner Priority Programme
NIHR: National Institute for Health Research
CLAHRC: Collaboration for Leadership in Applied Health Research and Care
NWC: North West Coast
CAMHS: Child and Adolescent Mental Health Service
LGBTQ +: Lesbian, gay, bisexual, transgender and queer (or questioning) and others
IAG: Information Advice and Guidance
