# Supplementary material for: An exploration of young people’s, parent/carers’, and professionals’ experiences of a voluntary sector organisation operating a Youth Information, Advice, and Counselling (YIAC) model in a disadvantaged area
Source: BMC Health Serv Res. 2022 Mar 23;22:383. doi: 10.1186/s12913-022-07800-1 (PMC8941750; doi:10.1186/s12913-022-07800-1)
Supplement: Supplementary file 1 — Additional file 1. [file 12913_2022_7800_MOESM1_ESM.docx]

**An exploration of young people's, parent/carers', and professionals' experiences of a voluntary sector organisation operating a Youth Information, Advice, and Counselling (YIAC) model in a disadvantaged area**

**Interview and Focus group topic guide (young people/ Parents)**

**1. Can you tell me how you got involved or started to come to YPAS as an organisation?**

Sub questions/prompts:

 How did you hear about YPAS?

 Can you tell me how you were referred to YPAS? Tell me what your experience was of the support offered?

 Can you tell me what information you were given about YPAS before accessing the service or what you knew about the service?

 Can you tell me what was or where your reasons for attending YPAS?

 How long did you have to wait for your appointment at YPAS? Can you tell me what would have supported you whilst waiting for your appointment at YPAS?

 Can you tell me what happened whilst you were waiting for your appointment at YPAS? Did anyone from YPAS make contact with you before your appointment? If so how did that make you feel? If not, how did that make you feel?

 Can you give us an example of any other support you accessed whilst waiting for your appointment at YPAS or anything you accessed before your referral to YPAS? (i.e. group, resource, website etc)

 Where you at crisis point when you arrived at YPAS? If yes – can you tell me a bit about why you were at crisis point and what the circumstances where in your life at this time?

**2. Can you tell me about the services you have accessed in YPAS and what you hoped to gain from accessing this service?**

Sub questions/prompts:

 What service/s have you accessed?

 What were the waiting times to access the service/s?

 Was the service individual to you or did it involve your family/wider network too?

 What information did you receive about the type of service you were accessing beforehand?

**3. Can you tell me what you know and think about YPAS as a service?**

Sub questions/prompts:

 Was it easy to access?

 What did you think about the location of the appointment, what would have made it better?

 What did you think about the time offered for your appointment, would anything have made it better?

 Did anyone from YPAS make contact with you before your appointment? If so how did that make you feel? If not, how did that make you feel?

 What do you think about the environment in YPAS? (was it welcoming, too clinical etc?)

 How did the staff at YPAS engage with you /and your family?

 What did you like about the service?

 What didn’t you like about the service?

**4. Can you tell me if YPAS has helped you and if so how has it helped you. If not can you tell me why you think this is and what would have helped or made it better?**

Sub questions/prompts:

 Has your mental health improved through attending YPAS and if so how? If not why do you think it hasn’t?

 Has YPAS helped improve your quality of life and if so how? If not why do you think that is?

 Did you feel involved in your care at YPAS and listened to?

 Has accessing YPAS resulted in you accessing any other services? If so what services are these and where are they?

**5. How did you find your experience of accessing YPAS?**

Sub questions/prompts:

 Did you feel involved in your care?

 Did you feel listened to?

 Did you have a choice in what service you accessed, when and where?

 Was the service friendly? If so how did this make you feel? If not how did this make you feel? What would have made it better?

 Did you feel supported? If so how? If not what would have made it better?

 Where your expectations met?

 How long did you have to wait to see someone?

**6. Is there anything else you would like to tell me about your time at YPAS?**

**Thank you for your time**

**An exploration of young people's, parent/carers', and professionals' experiences of a voluntary sector organisation operating a Youth Information, Advice, and Counselling (YIAC) model in a disadvantaged area**

**Interview and Focus group topic guide (professionals)**

1. **Can you tell me how you found out about YPAS as an organisation for 14-25 year olds?**

Sub questions/prompts:

- How did you hear about YPAS and what did you know about this organisation before making a referral or signposting individuals to it?
- Can you tell me how you referred to YPAS and what you think of the referral process?
- Can you tell me what information you were given about YPAS before referring to the organisation and how you accessed it?
- Can you tell me what was or where the reasons for making referrals to YPAS?
- Can you tell me if you have ever signposted young people or families to YPAS for self referral and if so how this went?
- Can you tell me about the communciation between you and YPAS before and after referral? Did you receive any consultation and support pre-referral, during or after referral?
- Can you tell me about any referrals made that were not accepted and what were the channels of communciation from YPAS about this?
- Can you tell me if or how the individual/s were communicated with during and following a referral to YPAS?

1. **Can you tell me about the services you have referred to in YPAS for 14-25 year olds and their families and what your expectations were of this/these service/s?**

Sub questions/prompts:

- What service/s have you referred to or accessed in YPAS for 14-25 year olds?
- Do you know what were the waiting times to access the service/s at YPAS following your referral?
- Was the refferral made for an individual or for the wider family/wider network too?
- What information did you receive about the type of service you were referreing to beforehand?
- Can you tell me about any communication from YPAS that took place betweent he individual/s referred? Was it positive or negative? What could have made it better?

1. **Can you tell me what you know and think about YPAS as an organisation for 14-25 year olds and their families?**

Sub questions/prompts:

- Can you tell me how accessible the organisation is for those who you have referred or accessed it?
- What did you think about the location of the appointments for those you have referred, what would have made it better?
- What did you think about the time offered for appoitnments for those referred, would anything have made it better?
- Can you tell me what the communciation was like from YPAS following referral and during the support offered to the individual/s you referred or accessed the service?
- What do you know about the environment in YPAS? (is it welcoming, too clinical etc?)
- What did/do you like about the organisation?
- What don’t you like about the organisation?

1. **Can you tell me if YPAS has helped the individual/s (14-25 year olds) you have referred and if so how has it helped. If not can you tell me why you think this is and what would have helped or made it better?**

Sub questions/prompts:

- What feedback have you had from the indiviudal/s you have referered to YPAS?
- Are you aware if the individual/s you have referred have been involved in their care at YPAS and listened to?
- Has accessing YPAS resulted in you referering again to this organisation or to any other services? If so what services are these and where are they? Did YPAs support with these other referrals?

1. **How did you find your expereince of referrering a 14-25 year old to YPAS and accessing any support offered?**

Sub questions/prompts:

- Did you feel involved, listened to and communicated with?
- Was the organisation friendly? If so how did this make you feel? If not how did this make you feel? What woud have made it better?
- Did you feel supported? If so how? If not what would have made it better?
- Where your expectations met?
- How long did you have to wait to speak to someone?

1. **Is there anything else you would like to tell me about YPAS as a servcie for 14-25 year olds?**

**Thank you for your time**
